# Experimental and clinical evidence in favour of an effective immune stimulation in ER-positive, endocrine-dependent metastatic breast cancer

**DOI:** 10.3389/fimmu.2023.1225175

**Published:** 2024-01-25

**Authors:** Andrea Nicolini, Giuseppe Rossi, Paola Ferrari

**Affiliations:** ^1^ Department of Oncology, Transplantations and New Technologies in Medicine, University of Pisa, Pisa, Italy; ^2^ Epidemiology and Biostatistics Unit, Institute of Clinical Physiology, National Research Council and Gabriele Monasterio Foundation, Pisa, Italy

**Keywords:** ER+ advanced breast cancer, cytokines, immunotherapy, cyclin kinase 4/6 inhibitors, TILs, anti-tumour immune response, cancer-immunity cycle

## Abstract

In ER+ breast cancer, usually seen as the low immunogenic type, the main mechanisms favouring the immune response or tumour growth and immune evasion in the tumour microenvironment (TME) have been examined. The principal implications of targeting the oestrogen-mediated pathways were also considered. Recent experimental findings point out that anti-oestrogens contribute to the reversion of the immunosuppressive TME. Moreover, some preliminary clinical data with the hormone-immunotherapy association in a metastatic setting support the notion that the reversion of immune suppression in TME is likely favoured by the G0-G1 state induced by anti-oestrogens. Following immune stimulation, the reverted immune suppression allows the boosting of the effector cells of the innate and adaptive immune response. This suggests that ER+ breast cancer is a molecular subtype where a successful active immune manipulation can be attained. If this is confirmed by a prospective multicentre trial, which is expected in light of the provided evidence, the proposed hormone immunotherapy can also be tested in the adjuvant setting. Furthermore, the different rationale suggests a synergistic activity of our proposed immunotherapy with the currently recommended regimen consisting of antioestrogens combined with cyclin kinase inhibitors. Overall, this lays the foundation for a shift in clinical practice within this most prevalent molecular subtype of breast cancer.

## 1 Introduction

Breast cancer is the most common cancer in women worldwide, with over two million new cases estimated in 2018 (1). Diagnostic and therapeutic advances have decreased the death rate. However, despite these advancements, metastatic disease develops in about 20% to 30% of patients (2). Sixty to eighty percent of all breast malignancies are ER+, HER2- luminal breast cancers, with an enhanced incidence in older age (3, 4). Despite ER+, HER2- luminal breast cancer having a better prognosis than other subtypes, distant metastases occur in more than 20% of patients. In recent years, the median overall survival (OS) rate of metastatic disease has increased, roughly ranging from two to four years (5, 6). In ER+, HER2- metastatic patients, endocrine therapy provides a clinical benefit ratio of 40% to 80%. However, endocrine resistance usually develops over time (3). Tumour infiltrating lymphocytes (TILs), tumour mutational load (TML) and PD-L1 expression are currently considered the main predictors of cancer immunogenicity. In ER+, HER2- luminal breast cancers, the lowest level of PDL1 expression (7–9), a lower TML (10–12) and a relatively low level of TILs have been reported in comparison with TNBC and HER2+ tumours. This led to consider ‘ER+’, including ER+, HER2-breast cancer as being immunologically ‘cold’ (13). Despite this, a relationship between oestrogen and inflammation in the TME has been reported (14–16), while there are findings that strongly support the concept that immunity and inflammation may be involved in the biology of this subtype as well (17). In 1992, we hypothesised that by inducing a G0-G1 state, anti-oestrogens in ER+, endocrine-dependent metastatic breast cancer counteracted tumour growth and the inhibition of the immune response promoted by oestrogen in the TME. Consequently, active immune stimulation with a beta-interferon interleukin-2 sequence could stimulate the effector immune cells to attack breast cancer cells. This hypothesis was initially supported by a pilot trial showing that progression-free survival (PFS) and OS were significantly prolonged in 26 studied patients compared with 30 historical controls (18). More recently (19), these findings were confirmed in a 2:1 ratio control-case observational study where 42 metastatic ER+ breast cancer patients were compared with 95 controls. In both clinical trials, the studied patients were treated with conventional anti-oestrogens concomitant with the beta-interferon interleukin-2 sequence, while controls received anti-oestrogens without immunotherapy. In the last decade, some experimental research has been providing support for our initial hypothesis. In this review, recent experimental and clinical findings, as well as current knowledge on the issue are examined. The keywords that can be used in searching the main scientific literature on the issue are ‘ER+ advanced/metastatic breast cancer’, ‘cytokines’, ‘immunotherapy’, ‘cyclin kinase 4/6 inhibitors’, ‘TILs’ ‘cancer immunity cycle’ and ‘anti-tumour immune response’. The principal current immunological therapies in ER+ metastatic breast cancer, including cyclin-dependent kinase (CDK) 4/6 inhibitors combined with anti-oestrogens, are also summarised. Thereafter, based on accumulated data, a mechanistic rationale is provided, pointing out that ER+ breast cancer is a molecular subtype where stimulation of effector immune cells concomitant with hormone therapy is a valid choice to hormone therapy alone.

## 2 Experimental findings on tumour growth and/or immune evasion of ER+ breast cancer cells and other cells in tumour microenvironment

### 2.1 Mechanisms related to breast cancer cells

During carcinogenesis, concomitant with the mitogenic-promoting role through ERs interactions of ER+ breast cancer cells, immunoediting occurs. Immune editing is usually reported to be a process by which tumour antigenicity changes due to the selective pressure of effector immune cells; although immunoediting may precede immune-evasion, they can be independent each other (20–22). The principal immune-evasive mechanisms include a down-regulation of antigen presentation, lack of immune effector and/or an increase in immune suppressor cells, as well as an up-regulation of checkpoint molecules (23). It is well known that tumour cells can be recognised by T cells if tumour-associated antigens (TAAs) are joined with human leukocyte antigen (HLA) molecules expressed on the surface of tumour cells or antigen-presenting cells. In about 30–40% of higher-grade breast cancers, classical HLA-A, HLA-B and HLA-C molecules, which are necessary to activate CD8+ T cells, are down-regulated; instead, the non-classical HLA-E, HLA-F and HLA-G molecules favouring the immune escape are upregulated. In one study, HLA class I down-regulation occurred in 64% of 141 ER+ breast cancer patients (24). In another study, HLA-F expression was found in 50% of 56 ER+ breast cancers (25). In another research, breast cancer biopsies from 52 patients with invasive ductal carcinoma and unselected for molecular sub-types suggested that the down-regulation of HLA-Ia, HLA-E and HLA-DR and the up-regulation of HLA-G and HLA-DQ by different processes were likely responsible for immune evasion and breast cancer aggressiveness (26). In an experimental study, MCF7 breast cancer cell lines carrying miR-18a hyper-expression and cultured with the THP-1 cell line, a human monocytic cell line derived from acute monocytic leukaemia, showed lower antigen presentation capability, higher differentiation of pro-tumorigenic M2 macrophages and increased invasiveness. In these cells, TAP -1, a crucial protein for antigen presentation, was again expressed following the inhibition of the Wnt pathway. Accordingly, in miR-18a high ER+ tumours, even though there was a dense lymphocyte infiltrate, a higher CD4+/CD8+ ratio and the M2 macrophage marker CD206 contemporaneously with the invasive marker MMP9 also occurred (27). A decreased rate of TILs has been reported in ER+ breast cancer. This may be due to the ER expression, which has been reported to both promote a Th2 immune environment and reduce MHC class II molecules in breast cancer cells (28). Besides HLA-A, antigen peptide transporter 1 and 2 protein expression (TAP1/TAP2), which are members of the ATP-binding cassette transporter family, are also down-regulated in high-grade breast cancer. This was found in 53 patients with breast carcinomas unselected for molecular subtypes (29) and in about 40% of metastatic lesions (30). Molecules involved in antigen presentation and interferon (IFN) response gene mutations account for another mechanism of immune evasion. For example, resistance to checkpoint blockade (31, 32) and other immune therapies can be due to mutations in beta2-microglobulin (B2 M), an element of MHC class I, and JAK1/2 kinases, downstream of IFN receptors. In particular, *JAK2* and *STAT3* were found to be significantly mutated in the metastasis/relapse screen, and all mutations in a cohort of 163 patients arose in ER+ breast cancers (33). CD16+ lymphocytes inversely correlated with the proportion of regulatory CD4+CD25+CD127- cells and the Ki-67 rate in tumour cells. Accordingly, a higher level of Ki-67 occurred concomitantly with decreased effector lymphocytes (CD8+ and CD16+) and a high percentage of regulatory CD8+CD11b-CD28- T cells (34). In addition, oncogenic pathway alterations are likely to play a crucial role in T cell cutting out or the inhibition of T cell activity (21). In particular, in breast cancer, PI3K pathway driver mutations involve 49% of luminal A and 7% of basal-like molecular subtypes. These driver alterations could contribute to immune-evasion or an immunosuppressive microenvironment (35), as well as to heterogeneity with respect to TILs in the breast cancer subtypes. Tumour cells can constitutively express indoleamine-pyrrole 2,3 dioxygenase (IDO) or up-regulate it in response to interferon-gamma and oestrogen receptor signalling, and IDO over-expression results in diminished local anti-tumour response (36–38). IDO has been established as a normal mechanism of peripheral tolerance and immuno-suppression, and high IDO levels have been found in ER+ breast cancer compared to ER- tumours. In ER+ tumours, it was higher in more advanced stages (39, 40). In an experimental mouse model of luminal breast cancer, CCL5 also named RANTES (regulated on activation, normal T cell expressed and secreted) expression directly correlated with tumour progression. Moreover, with a deeper analysis, it was uncovered that high tumour CCL5 levels induced the polarisation of CD4+ T cells toward a pro-tumorigenic Th2 phenotype (17). Taken together, these data suggest that in hormone receptor positive breast cancer, the interaction between tumour cells and the immune microenvironment occurs differently than in other breast cancer subtypes, involving the intercommunication of endocrine factors with pro-inflammatory status and immune cells modulated by the TME (17).

### 2.2 Mechanisms related to other cells

Cellular components of the TME usually include tumour-associated macrophages (TAMs), mesenchymal stem cells (MSCs) and/or cancer-associated fibroblasts (CAFs), endothelial cells, immune cells such as T and B cells, natural killer (NK) cells and myeloid-derived suppressor cells (MDSCs) (41).

#### 2.2.1 Cancer-associated fibroblasts and mesenchymal stem cells

The CAFs are among the most prevalent stromal cell types within the TME and in primary breast cancer tissue express ER-alpha (42). In addition, the E2 responsive gene, liver receptor homolog-1 (LRH-1), is up-regulated in CAFs compared to normal fibroblasts (42). LRH-1 also directly regulates the aromatase encoding gene, CYP19A1 (43). The CAFs promote tumour growth and progression in various ways: increasing oestradiol (E2) levels, secreting various factors (hepatocyte growth factor (HGF), transforming growth factor (TGF)-beta, stromal cell-derived factor (SDF)-1, vascular endothelial growth factor (VEGF)) and matrix metalloproteinases (MMPs), inducing stemness, epigenetic changes and/or endothelial to mesenchymal transition (EMT). The CAFs can promote angiogenesis and, by delivering IL1, IL6 and TGF-beta, the immune suppression (44, 45). IL-6, in particular, is a pro-inflammatory cytokine that is known to increase ER+ breast cancer cell growth and invasion. In a study, a direct correlation between mesenchymal stem cell (MSC)-related genes and PD-L1 expression in different molecular subtypes of breast cancer occurred likely through the secretion of various cytokines, especially CCL5 (46). In an experimental study in oestrogen receptor-alpha (ER-alpha)-positive human breast tumour cell lines (MCF-7, T47D, BT474, and ZR-75-1), all cell lines had low basal activation of the signal transducer and activator of transcription 3 (STAT3); however, chronic phosphorylation of STAT3 on tyrosine-705 in all the tumour cell lines was observed after they were exposed to MSC. MCF-7 growth rates increased more than twice when co-cultured with MSC *in vitro*, and there was even higher growth *in vivo* concomitant with autocrine IL-6 production (47). These findings were confirmed in another experimental study in mice, where local tumour-associated fibroblasts (TAFs) provided a paracrine production of high levels of IL-6 that induced STAT3 activation and ER-alpha+ tumour cell proliferation (48).

#### 2.2.2 Tumour-associated macrophages

M1 and M2 are the two macrophage phenotypes, each corresponding to a different and largely opposite function. M1 macrophages secrete IFN, interleukin 12 (IL-12) and TNF, which are pro-inflammatory cytokines and contribute to tumour rejection and antigen presentation (49). On the other hand, M2 macrophages secrete interleukins 4, 5, 6 and 10 (49), also known as type-2 cytokines—all of which favour tumour cell growth and immune evasion (50). Besides, TAMs from breast cancer may express the aromatase enzyme that allows E2 production within the TME, which, in turn, promotes ER+ breast tumour cell proliferation (51). In an investigational report, brain metastases of pre-menopausal breast cancer patients were largely infiltrated by M2 microglia, and the same occurred *in vivo* after mice were systemically treated with oestrogen. Oestrogen-signalling inhibition either by tamoxifen or surgical resection of mice ovaries impeded M2 microglial polarisation, reduced the secretion of C-C motif chemokine ligand 5 and suppressed brain metastasis (52). As demonstrated in an ER+ breast cancer murine model, oestrogens can induce TAM M2 polarisation and infiltration. In fact, in the studied murine model, tumoral M2 TAM infiltration was increased by E2 treatment, unlike untreated controls, which showed M1 TAM infiltration (53). Moreover, vascular endothelial growth factor, a mediator of M2 macrophage recruitment, was also increased by E2 (53, 54). Tumour progression is also promoted by TAMs through their production of growth-promoting factors, and the increased invasiveness of ER+ breast cancer cell lines is induced via the TNF-alpha-NFkB pathway (17).

#### 2.2.3 Myeloid-derived suppressor cells

Most immune cells, including T-cells, B-cells and NK cells, express ER and PR; and with regard to ER, ERalpha 46 is the principal isoform (55). The MDSCs favour immune escape and tumour development (56). MDSCs expressing ER-alpha have been found in the tumour tissue, bone marrow and peripheral blood of human ovarian cancer patients, and ER capability to expand MDSCs was proven by the ER antagonist methylpiperidino pyrazole (MPP). This ER antagonist inhibited MDSC proliferation *in vitro* (57). In ER+ breast cancer, ELF5 and CCL2 over-expression likely account for higher recruitment of MDSCs, while the immune-suppressive activities of MDSCs are promoted by an increase in IFN signalling (58). In ovarian tumour–bearing mice, the administration of oestrogens induced STAT3-signaling hyperactivation, which governs myeloid differentiation and development (59) through transcriptional JAK2 and SRC up-regulation and enhanced activity (57). The same occurred in lung and breast cancer murine models and the E2-dependent tumour growth was inhibited by MDSC depletion with anti-Gr1 antibodies. ER signalling enhanced the progression of some oestrogen-insensitive tumour models both by inducing the mobilisation of MDSCs and their inherent immunosuppressive capability *in vivo* (57).

#### 2.2.4 CD4+ T cells

Tumour-growth inhibition and IFN and IL-12 increase join with T helper 1 (Th1) T cell responses, while T helper 2 (Th2) responses are associated with elevated IL-4, favouring tumour progression (60, 61). Studies both in murine models and humans report that high oestrogens account for Th2-enhanced responses (62) and IL-4 production increase (63, 64). In ER- compared to ER+ breast tumours, elevated cytotoxic T lymphocytes (CTLs), Th1 T cells and B cells were reported (65). Moreover, in this study, as previously reported, ER activity negatively correlated with tissue breast cancer infiltration of each of these immune cells (65, 66).

#### 2.2.5 Cytotoxic T cells (CTLs) and natural killer (NK) cells

An active granules secretion, also known as granule-mediated exocytosis, is one modality by which these cells kill pathogenic and tumour cells (67). These granules contain serine protease granzymes, among them granzyme B, which, after entering into the target cells, are responsible for caspase-dependent apoptosis (68). However, an over-expression of the granzyme B inhibitor, proteinase inhibitor-9 (PI-9), which avoided the NK and CTL-induced apoptosis was found when ER+ expressing human liver carcinoma cells were cultured with E2 (69). This also occurred in ER+ MCF7 breast cancer cells where, after oestrogen treatment, the E2-induced PI-9 expression avoided the apoptosis of cancer cells, while PI-9 knockdown allowed the NK granule-mediated apoptosis (70). These findings suggest that oestrogens increase immune evasion by inhibiting NK and CTL-mediated tumour cell apoptosis.

#### 2.2.6 FoxP3 expressing regulatory T cells (Tregs)

Tregs are recruited by CCL5 and CCL22, which are produced by CD8+ T cells and DCs. Physiological doses of E2 administered to immunocompetent ovariectomised mice enhance CD4+CD25+ Treg expansion and up-regulate Foxp3 expression. Furthermore, ER+ CD4+CD25- T cells treated with oestrogens transform into CD4+CD25+ Treg phenotype and inhibit T cell proliferation *in vitro* (71). Foxp3 expression induced by oestrogens in murine Tregs has been also reported. Foxp3 plays a crucial role in Treg function, and tumoral tissue FoxP3+ Tregs infiltration predicts poor prognosis in many different cancers (72, 73), including ER+ breast cancer (74). In a study, osteoclasts differentiation and bone resorption were suppressed from Treg cells by IL-10 and TGF-beta1 secretion. In the same study, an induced IL-10 and TGF-beta expression in Tregs occurred when Treg and blood mononuclear cells co-cultures were treated with oestrogen. Therefore, the authors concluded that oestrogen enhances Tregs effects on osteoclasts through increased secretion of these two cytokines from Tregs (75). A significant reduction of FoxP3+ Tregs in ER+ breast tumours occurred after patients had received letrozole administration (76). Besides, PD-1 up-regulation and increased suppressive activity were observed in Tregs isolated from mice and treated with oestrogen, while ER knockout reduced Treg suppression and PD-1 expression (77).

#### 2.2.7 Immune-suppressive cancer-associated fibroblasts, tumour-associated macrophages, myeloid-derived suppressor cells, and FoxP3-expressing regulatory T cells

In breast cancer, including the ER+ molecular subtype, MDSCs, M2 macrophages and the other immune suppressor cells support tumour growth and metastasisation and inhibit T lymphocytes and NK cells through IDO, IL10, reactive oxygen species (ROS), nitric oxide (NO) and other suppressive molecules (78, 79). Therefore, an increase in MDSC, M2 macrophages, Tregs and CAFs may lead to the inhibition or decrease of CD8+ T cells activity (80). Additionally, in regional lymph nodes, a small population of IDO-expressing plasmacytoid DCs (pDCs) may inhibit effector T cells and activate T regulatory cells (81–83). The main mechanisms of immune evasion in ER+ breast cancer mediated by breast cancer cells, stromal cells (CAFs and/or MSCs and TAMs) and immune cells are summarised in **Tables 1A**, **B**.

**Table 1A:** Main mechanisms of immune suppression (I.S.)* in ER positive breast cancer.

Target cell	Mechanism	Outcome	Ref (N)
BCCs	Downregulation of classical HLA-I and HLA-II as well as upregulation of non-classical HLA-E, HLA-F, HLA-G and HLA-DQ molecules	Immune evasion	(24–26)
	Mutations in antigen presentation and INF response genes (beta2-microglobulin, JAK2/STAT3)	Immune evasion	(31–33)
	Oncogenic pathways alterations (PI3K)	T cell exclusion or compromised T cell activity	(35)
	Constitutive or through INFgamma and ERalpha signalling-IDO overexpression	Peripheral tolerance and I.S.	(36, 37, 40)
	High CCL5 levels	Th2 phenotype promotion	(17)
*TME stromal cells*			
CAFs	E2 production within TME; ERalpha expression and LRH1E2 responsive gene upregulation; IL-1, IL-4, IL-6, TGFbeta, VEGF, HGF, SDF-1, MMPs secretion	EMT and/or angiogenesis, I.S., tumor growth and invasion	(42–45)
MSCs	STAT3 activation through IL-6 production	Tumor cell growth and invasion	(47, 48)
TAMs	E2 production within TME; E2-induced M2 phenotype; secretion of type-2 cytokines including IL-4, IL-5, IL-6, IL-10 and of growth promoting factors including VEGF; autocrine loop sustained by E2 increased VEGF; promoted TNFalpha-NFkB pathway	Tumor progression, invasiveness and I.S.; M2 recruitment by VEGF	(17, 49, 51, 53)

*It is inclusive of immune evasion and/or immune inhibition. BCCs, breast cancer cells; HLA, human leukocyte antigen; JAK, janus kinase family; STAT, signal transducer and activator of transcription; PI3K, phosphatidylinositol-3 kinase; CCL5, chemokine (C-C motif) ligand 5, also known as RANTES (regulated on activation, normal T cell expressed and secreted); IFN, interferon; Th, T helper cell; IDO, indoleamine-pyrrole 2, 3 dioxygenase; CAFs, cancer associated fibroblasts; MSCs, mesenchymal stem cells; TAMs, tumor-associated macrophages; E2, estradiol; TME, tumor microenvironment; LRH1, liver receptor homolog-1; IL, interleukin; TGFbeta, transforming growth factor beta; VEGF, vascular endothelial growth factor; HGF, hepatocyte growth factor; SDF1, stromal cell-derived factor-1; MMPs, metalloproteinases; EMT, epithelial to mesenchymal transition; M2, type 2 TAM; TNFalpha, tumor necrosis factor alpha; NFkB, nuclear factor k light –chain enhancer of activated B cells; also see tex.

**Table 1B:** Main mechanisms of immune suppression (I.S.)* in ER positive breast cancer.

Target cell	E2-induced mechanism	Outcome	Ref (N)
**Immune cells (TME)			
MDSCs	Expansion through recruitment by ELF5 and CCL2-expressing ER+ BCCs; enhanced immune suppressive activity by increased STAT3 signalling; MDSCs mobilization	I.S.	(57–59)
CD4+ T cells	Th2 response and upregulation of IL-4 production	I.S.	(62–64)
CD8+ T cells (CTLs) and NK cells	Upregulation of PI-9	Inhibition of CTLs and NK mediated tumor cell apoptosis	(69, 70)
CD4+CD25+ FOX-p3 cells (Tregs)	Treg expansion and upregulation of FOX-p3 expression; recruitment by CCL5 and CCL22 expressing CD8 T cells and DCs; increased PD-1 expression and suppressive activity	T cell proliferation inhibition; suppression of the effector immune cells activation	(71, 77)
CAFs, M2, MDSCs, Tregs	Release of IL-10, IDO, ROS, NO suppressive mediators	I.S.	(78, 79)
APCs	Inhibitory activity; downregulation of their priming function, upregulation of immunosuppressive molecules and secretion of immunosuppressive cytokines by mutual interactions with Tregs	I.S.	(81–83)

*It is inclusive of immune evasion and/or immune inhibition. **Most of them express ERs, mainly ERalpha 46. E2, estradiol; TME, tumor microenvironment; MDSCs, myeloid-derived suppressor cells; ELF-5, E74-like factor 5; CCL, C-C motif chemokine ligand; STAT, signal transducer and activator of transcription; Th, T helper cell; IL, interleukin; PI-9, proteinase inhibitor-9; CTLs, cytotoxic T lymphocytes; FOX-p3, forkhead box p3; DCs, dendritic cells; CAFs,cancer associated fibroblasts; M2, type 2 macrophage; PD-1, programmed cell death protein 1; IDO, indoleamine-pyrrole 2,3 dioxygenase; ROS, reactive oxygen species; NO, nitric oxide; APCs, antigen presenting cells; also see text.

## 3 Chronic inflammation and high E2 levels are two mainstays in the induced immune suppression of the tumour microenvironment

### 3.1 Main mechanisms driven by chronic inflammation through cytokines production

Chronic inflammation is a well-recognised ancillary mechanism of cancer progression that joins common breast cancer risk factors such as menopause and obesity. The inflammatory TME milieu promotes a more aggressive, endocrine-resistant ER+ breast cancer phenotype (16, 17). IL-6, TGF-beta, TNF-alpha, NF-kB, COX-2 and PGE-2 are pro-inflammatory cytokines present in the TME of ER+ breast cancer that are capable to activate pro-tumoral pathways mediating proliferation, immune evasion and metastasis (16, 84). The principal pro-tumorigenic actions of IL-6 and TGF-beta cytokines have been just mentioned and reported (see **Table 1A**). TNF-alpha, which is a ubiquitous TME cytokine secreted by tumour cells and macrophages, may promote metastasis of breast and other cancers. In an experimental study, TNF-alpha enhanced the invasive capacity of MCF-7 cells through an increased expression of metastasis-related genes (85). TNF-alpha may also up-regulate aromatase expression in stromal cells and may play a principal role in governing oestrogen biosynthesis in adipose tissue (86). TNF-alpha strongly induces E2 increased levels in TME by maintaining the surrounding fibroblast in an undifferentiated state. In turn, oestrogens themselves up-regulate transcription and secreted levels of TNF-alpha in ER+ breast cancer cells, therefore creating an autocrine-positive feedback loop (87). TNF-alpha also mediates cancer-related inflammation through the activation of NF-kB (17). The role of NF-kB signalling in tumour initiation and inflammation is well established. Constitutive activation of NF-kB has been shown in various types of cancer; NF-kB leads to a release of adhesion molecules and immune-regulatory cytokines (such as TNF-alpha, IL-1, IL-6 and IL-8), which converge leukocytes to sites of inflammation (88). Significant correlations between ER-alpha, TNF-alpha and NF-kB expression have been shown in breast cancer tissues (89). A study showed that pre-metastatic niche formation in the lungs was promoted by PGE2 produced by fibroblasts through dysfunctional dendritic cells (DCs) and suppressive monocytes. This process was propagated by tumour inflammation, mainly by interleukin-1beta. Ablation of the Ptgs2 gene (encoding COX-2) in fibroblasts reversed the immune-suppressive phenotypes of lung myeloid cells in multiple breast cancer models (90). Moreover, it was reported that obesity-related breast inflammation is linked with high aromatase activity, likely through the promotion of CYP 19 transcription by an increase in cyclooxygenase 2 (COX2) derived prostaglandin-E2 (PGE2) (17). Overall, ER and aromatase expression in stromal and immune TME point out an important immune-modulatory role of ER signalling in cancer biology.

### 3.2 Oestrogens promote tumour growth and immune evasion in TME

**Figure 1:**
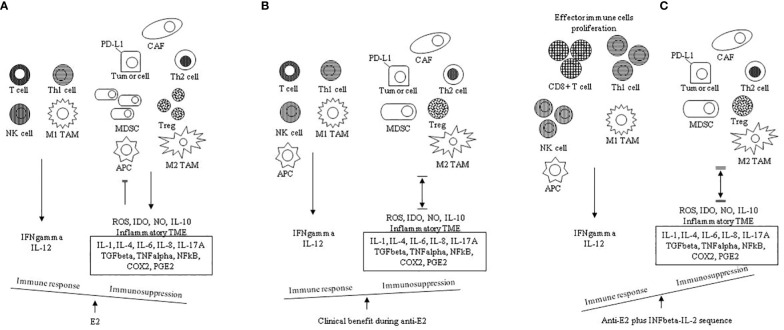
Chronic inflammation and high E2 are two mainstays in the TME: proposed mechanisms governing the immune balance. **(A)** in the absence of antiestrogens (anti-E2), the E2 pathway favors a tumor- promoting immunosuppressive TME shifting the balance in favor of Th2 responses and M2 TAM infiltration compared to Thl responses and M1 TAM infiltration with promotion of the associated cytokines (IL-1, IL-4, IL-6, IL-8, IL-10, IL-17A) and suppressive mediators (TGFbeta, TNFalpha, NFKB, COX2, PGE2). E2 further contributes to immunosuppression through proliferation of Tregs, expansion of MDSCs, induced inhibitory activity of APCs, increased tumor cell PD-L1 expression and inhibition of CD8+ T cell and NK cell induced apoptosis. TME inflammation is sustained by the co-inflammatory cytokines (IL-1, IL-4, IL-6, TNFalpha, NFKB). **(B)** anti E2 counteracts the immunosuppressive E2 actions (also see **Table 1B**); this shifts the balance by increasing the immune response and decreasing the immunosuppression. Immunosuppression is inclusive of immune evasion and immune inhibition; therefore, in ER positive breast cancer in clinical benefit during anti-estrogens, the tumor-promoting immunosuppressive TME is reverted |↔| **(C)** the addition of the INFbeta- IL-2 sequence boosts the effector immune response; this shifts the balance by further decreasing the immune suppression ‖↔‖ E2, estradiol; Thl, T helper type 1 cell; Th2, T helper type 2 cell; M1 TAM, type 1 tumor associated macrophage; M2 TAM, type 2 tumor associated macrophage; CAF, cancer associated fibroblast; MDSC, myeloid-derived suppressor cell, APC, antigen presenting cell; ROS, reactive oxygen species, IDO, indoleamine-pyrrole 2, 3 dioxygenase; NO, nitric oxide; IL, interleukin; TME, tumor microenvironment; TGFbeta, transforming growth factor beta; TNFalpha, tumor necrosis factor alpha; NFkB, nuclear factor k light-chain enhancer of activated B cells; COX2, cyclooxygenase-2; PGE2, prostaglandin E2.

It is well known that oestrogens favour the development and progression of some various cancers, including breast cancer (91). For an effective anti-cancer immune response, a series of stepwise events are involved. These steps have been defined as the cancer-immunity cycle (92, 93). In cancer patients, the cancer-immunity cycle is impaired, and many mechanisms facilitate immune evasion. Among them, T cells homing and infiltration of the tumour may be compromised, and/or more commonly, the effector immune cells might be suppressed by factors produced in the TME (94). It has been shown that in many cancer patients, immune system deficiency may be due to the presence of negative regulators of T cell responses (checkpoints) in lymph nodes and tumour tissues (immunostat function) (95). In particular, factors in the TME, including PD-L1 and PD-1 molecules, can regulate the response of activated anti-tumour T cells, acting as an immune rheostat or ‘immunostat’ (96, 97). This highlights that the cancer immune response depends on many carefully governed events; therefore, it may be addressed, at best, only if considered not as a simple but as a very complex process. Most functions of immune escape are E2/ERs induced by non-cancerous cells in the TME. It has been established that non-cancerous cells in the TME are involved in tumour progression, and the interaction among cancer cells, immune and stromal cells and extracellular mediators within the TME may influence the immune response to tumour and immunotherapy (21, 24, 98, 99). In particular, oestrogens may favour an immunosuppressive TME by enhancing Th2 responses, tumour-promoting cytokines (IL-4, IL-6, IL-17A and TNF) and M2 TAM infiltrations, unlike M1 TAM infiltration and Th1 responses, which are commonly joined with Th1 cytokines (IL-12 and IFN) (**Figure 1A**). Oestrogens induce the expansion of Tregs and MDSCs (57, 100, 101) as well as inhibitory activity of the antigen-presenting cells (APCs) (102). Particularly, it has been reported that when APCs are exposed to Tregs, their antigen-presenting function is down-regulated and the expression of immunosuppressive molecules and cytokines secretion are increased (103). *In vitro*, after E2 treatment, PD-1 ligand (PD-L1) up-regulation on ER+ endometrial and breast cancer cells through activation of the PI3K pathway has been observed (104).

Within tumours, T cell activation commonly up-regulates PD-1 expression, and PD1+ T cells are usually confined to tertiary lymphoid structures (TLS), which take part of the tumour stroma and are made by B and T cells (105). Cytotoxic T cell exhaustion and consequent immune escape derive from interactions between PD-L1+ tumour cells and PD-1+ T cells (106). Additionally, CAFs may favour a pro-tumour environment by paracrine secretion of E2 and IL-6. Oestrogens, through non-cancerous cells comprising the TME mediated actions and promoting angiogenesis and cytokines release in the TME, further promote tumour growth, combined with immune evasion. In conclusion, the evidence that E2 largely regulates the immune TME suggests an investigation into the reversal of tumour immune evasion by addressing TME as another beneficial target of anti-oestrogen therapy.

The principal mechanisms of tumour progression and immune suppression promoted by chronic inflammation and increased oestrogens in TME are summarised in **Table 2** and **Figure 1**. In particular, in the absence of anti-oestrogens, the E2 pathway prevails, and an immunosuppressive TME is favoured. Th2 responses and M2 TAM infiltration with the associated suppressive cytokines (IL-1, IL-4, IL-6, IL-8, IL-10, IL-17A) and suppressive mediators (TGF-beta, TNF-alpha, NFkB, COX2, PGE2) overcome Th1 responses and M1 TAM infiltration. E2 further contributes to immune suppression through the proliferation of Tregs, expansion of MDSCs, induced inhibitory activity of APCs, increased tumour cell PD-L1 expression and the inhibition of CD8+ T cell and NK cell-induced apoptosis (**Figure 1A**). When the immunosuppressive E2 actions are counteracted by anti-oestrogens (also see **Table 1B**), the immune response increases while the concomitant immune suppression decreases; thus, in ER+ breast cancer in clinical benefit during anti-oestrogens, the common immunosuppressive TME can be reverted (**Figure 1B**). Finally, the addition of the INF-beta-IL-2 sequence boosts the effector immune response, increasing the immune stimulation that shifts the balance by further decreasing the immune suppression (**Figure 1C**).

**Table 2:** Main mechanisms of tumor progression and immune suppression (I.S.)* driven by chronic inflammation and increased E2 levels in ER positive breast cancer TME.

Inflammatory cytokine	Mechanism	Outcome	Ref (N)
IL-6	See **Table 1A**, CAFs, MSCs and TAMs	See **Table 1A**, CAFs, MSCs and TAMs	–
TNFalpha	Major mediator of cancer-related inflammation via NFkB activation; regulation of the expression of metastatic promoting genes in ER positive BCCs (see **Table 1A**, TAMs); induced E2 production in TME; autocrine loop in ER+BCCs sustained by increased TNalpha-E2 production	Promotion of tumor progression and metastatic phenotype	(16, 17, 85, 87)
COX2, PGE2	Associated with high aromatase activity and enhanced E2 synthesis through induced CYP19 transcription	Tumor growth and I.S.	(16, 17)
TGFbeta	See **Table 1A**, CAFs	See **Table 1A**, CAFs	–
NF-kB	TNFalpha, IL-1, IL-6, IL-8 and adhesion molecule induction	Regulation of immune response; leukocytes recruitment (see **Table 1A**, TAMs)	(88)
*E2*			
Increased E2 levels	Promoted genomic and non-genomic pathways and angiogenesis; ERalpha mediated mechanism of immune evasion (see **Table 1A**, BCCs); upregulation of transcription and secreted levels of TNFalpha in ER positive BCCs (autocrine positive feedback loop); ER-alpha expression and modulation in stromal cells; induction of Th2 response and M2 polarization (see **Tables 1B**, CD4+ Tcells and **Table 1A**, TAMs respectively) with related cytokine production; expansion of Tregs, MDSCs and APCs induced-inhibitory activity, increased tumor cell PD-L1 expression (see **Table 1B**, Tregs, MDSCs and APCs respectively)	Tumor growth and I.S.	(91, 100–102, 104)

*It is inclusive of immune evasion and/or immune inhibition. E2, estradiol; TME, tumor microenvironment; TNFalpha, tumor necrosis factor alpha; NFkB, nuclear factor k light –chain enhancer of activated B cells; COX2, cyclooxygenase-2; PGE2, prostaglandin E2; CYP19; aromatase or estrogen synthetase; Th2, type 2 T helper cell; IL, interleukin; ER, estrogen receptor; BCCs, breast cancer cells; M2, type 2 macrophage; MDSCs, myeloid-derived suppressor cells; APCs, antigen presenting cells; PD-L1, programmed cell death ligand 1; also see text.

## 4 Immunological therapies and cyclin-dependent kinase 4/6 inhibitors combined with anti-estrogens in ER+ metastatic breast cancer

Despite of the increasing interest in immunotherapy against cancer, in breast cancer, only anti-HER2 monoclonal antibodies and PD-L1 inhibitors combined with conventional chemotherapy are used in current clinical practice in HER2+ (107, 108) and triple-negative breast cancer patients (TNBC), respectively (109, 110).

In the previous years, mostly in the nineties and early two thousand, a few investigational attempts of immunotherapy in advanced breast cancer, including the ER+ subtype, were carried out based on the use of cytokines, mainly interleukin-2 or interferons alone or with anti-oestrogens. However, despite some occasional favourable results, this research, which we have widely reported in a review article (111), has been abandoned early after. More recently, the safety and anti-tumour activity of some PD1/PD-L1 inhibitors in hormone receptor-positive advanced or metastatic breast cancer patients have been evaluated in a few investigational clinical trials. The phase Ib KEYNOTE-028 (8) and the phase II Kelly (112) trials, conducted with pembrolizumab (PD1 inhibitor), enrolled heavily pre-treated ER+, HER2- advanced breast cancer patients selected or not for PD-L1+ tumours, respectively. In the former, where pembrolizumab was given alone, the overall response rate (ORR) was 12% and the median duration of response was 12 months; hence, the authors concluded that in the evaluated population, pembrolizumab was well tolerated with a modest but prolonged ORR (8). In the latter, where pembrolizumab was given in combination with eribulin, the ORR, PFS and clinical benefit ratio were 40.9%, 6 months and 56.8%, respectively (112). In another trial, which was a phase I dose escalation study, the safety of tremelimumab (CTLA-4 inhibitor), given on the third day of palliative RT, was evaluated in five patients with hormone receptor positive metastatic breast cancer. The authors concluded that there was a need to optimise this combination approach (113). Apart from these preliminary attempts, ER+ luminal breast cancer is considered immunologically ‘cold’ (13) and unsuitable for immunological therapy. Despite this, the aforementioned experimental findings demonstrate that oestrogen signalling modulates the immune TME through enhanced pro-tumoral responses. By reversing an immunosuppressive TME, anti-oestrogen therapy has the potential to increase the response to immunotherapy in endocrine-dependent breast tumours. This seems to confirm our initial hypothesis that combining anti-oestrogens with an immunotherapy, stimulating the effector immune cell is a rational research field in ER+, endocrine-dependent metastatic breast cancer patients. Consistently, in 2005, we first reported twice (18, 114) the successful immune stimulation obtained with beta-interferon interleukin-2 sequence in an open pilot study recruiting 26 metastatic breast cancer patients in a state of clinical benefit during first-line hormone therapy with tamoxifen. Successively, from 2007 to 2019, the findings of this pilot study were updated more times (115–118). These 26 patients were compared with 30 historical controls from the same Centre and literature data. All the controls had received only anti-oestrogens. A significant median PFS and OS increase was found in the 26 studied patients. These findings were successively confirmed in two reports of a 2:1 ratio control-case observational study in which 95 ER+, HER2- controls were compared with 42 ER+ cases, both recruited from the same oncologic Centre (19, 119). Most controls were ER+, HER2- patients. We also reported on the potential mechanistic rationale of the successful manipulation (120). Currently, in ER+, HER2- metastatic breast cancer patients, CDK 4/6 inhibitors in addition to anti-oestrogens, usually aromatase inhibitors (AIs), are recommended as the first-line salvage treatment (121–124). These drugs inhibit the G1/S phase, mainly reinforce the anti-proliferative action induced by anti-oestrogens and have been recently investigated in early randomised clinical trials. In particular, ribociclib (121), palbociclib (122, 123) and more recently, abemaciclib (124) have shown significant prolongation of median PFS compared to AIs alone. **Table 3** summarises the main characteristics and results of clinical studies conducted with our proposed immunotherapy in addition to anti-oestrogens and of clinical trials carried out with CDK 4/6 inhibitors in addition to AIs in first-line ER+, HER2- metastatic breast cancer patients.

**Table 3:** Main characteristics and outcomes in clinical trials carried out with antioestrogens plus cyclin kinase inhibitors (CKi) and in our clinical studies with antioestrogens (ae.) plus immunotherapy (HIT) in first line endocrine dependent metastatic breast cancer patients.

Clinical trial	Intervention	Main characteristics												Outcome			Ref
		Pts (N)		Type of ae.		Study arm		Control		Study arm		Control		PFS (mo)	OS (mo)	G3-4 AEs	
		Study arm	Control	Study arm	Control	ER+	HER2-	ER+	HER2-	LA/RC	M	LA/RC	M				
Monaleesa-2 (phase III)	Ribociclib plus ae. *vs.* ae.	334	334	Let (AI)	Let (AI)	332 (99.4%)	334 (100%)	333 (99.7%)	333 (99.7%)	1 (0.3%)	333 (99.7%)	3 (0.9%)	331 (99.1%)	16 *vs.* 25.3	63.9 *vs.* 51.4	>10%	(121, 125)
Paloma-2 (phase III)	Palbociclib plus ae. *vs.* ae.	444	222	Let (AI)	Let (AI)	84 (100%)	84 (100%)	81 (100%)	81 (100%)	3 (4%)	81 (96%)	1 (1%)	80 (99.4%)	14.5 *vs.* 27.6	53.9 *vs.* 51.2	>15%	(122, 123)
Monarch-3 (phase III)	Abemaciclib plus ae. *vs.* ae.	328	165	Let/Ana	Let/Ana	328 (100%)	328 (100%)	165 (100%)	165 (100%)	0	328 (100%)	0	165 (100%)	14.8 *vs.* 28.2	NA	58%	(124)
Pilot study	INF-beta-IL-2 plus ae. *vs.* ae.	26 (ref. 18) 29 (ref. 114)	30	Tam (13 pts) Tor (13 pts)	Tam (30 pts)	14 (54%)	NA	12 (40%)	NA	0	26 (100%)	0	30 (100%)	16 *vs.* 38	31 *vs.* 103	0 (0%)	(18, 114)
2: 1 case control observational study	INF-beta-IL-2 plus ae. *vs.* ae.	42	95	Tam (27 pts) Tor (12 pts) AI (3 pts)	Tam (5 pts) Fulv (12 pts) AI (78 pts)	27 (64%)	26 (59.5%)	95 (100%)	91 (95.8%)	0	42 (100%)	0	95 (100%)	18 *vs.* 31	62 *vs.* 81	0 (0%)	(19, 119)

HIT, hormone-immunotherapy; LA/RC, locally advanced/recurrent disease; M, metastatic; INF, interferon; IL-2, interleukin-2; PFS, progression free survival; OS, overall survival; AE, adverse event; Tam, tamoxifen; Tor, toremifene; AI, aromatase inhibitor; Fulv, fulvestrant; Let, letrozole; Ana, anastrozole; NA, not available.

## 5 Mechanistic rationale of our proposed immunotherapy

Using *in-vivo* experimental models (126, 127), it was found that anti-oestrogens prolonged the G0-G1 state (resting state) of ER+ cancer cells and favoured a cytostatic rather than a cytotoxic-cytocidal effect. Besides, some further experimental studies conducted in human breast cancer cell lines provided the grounds to combine anti-oestrogens with immune therapies, including interleukin-2 in breast cancer patients (128, 129). However, in 1992, at the beginning of our open pilot clinical trial, neither the capability of the cell-mediated immune system to recognise surface TAAs and promote an immune response had been proven nor most mechanisms of ER-mediated immune suppression in the TME had been elucidated. Despite this, we assumed both of them based on preliminary clues while the reasons for the administration of the beta-interferon interleukin-2 sequence and their doses were well documented and described in our first report (18).

### 5.1 Conventional anti-estrogens therapy inhibits tumour growth and reverses the immunosuppressive tumour microenvironment

Over the past three decades selective oestrogen receptor modulators (SERMs) or down-regulators (SERDs) and AIs, that inhibit E2 signalling, have strongly decreased breast cancer mortality (130). Welboren et al. (131), confirming other previous findings by Frasor et al. (132, 133), reported that tamoxifen antagonised most of the E2-up-regulated genes at the same time possessing agonistic behaviour on E2-downregulated genes (134). Moreover, many other experimental studies have reported that mechanisms promoting at the cancer cell level, tumour growth often join with the inhibition of the immune response (135). Based on this, it can be inferred that anti-oestrogen therapy has the potential not only to inhibit tumour growth and progression through the anti-proliferative action mediated by ERs via the ‘genomic’ and ‘non-genomic’ pathways (136), but also to favour the immune response in E2-sensitive tumours by the reversion of an immunosuppressive TME (137) (**Figure 1B**).

### 5.2 Laboratory data: beta-interferon interleukin-2 sequence boosts the innate and adaptive immune response in metastatic ER+ breast cancer patients in a state of clinical benefit (G0-G1 state) during hormone therapy

**Figure 2:**
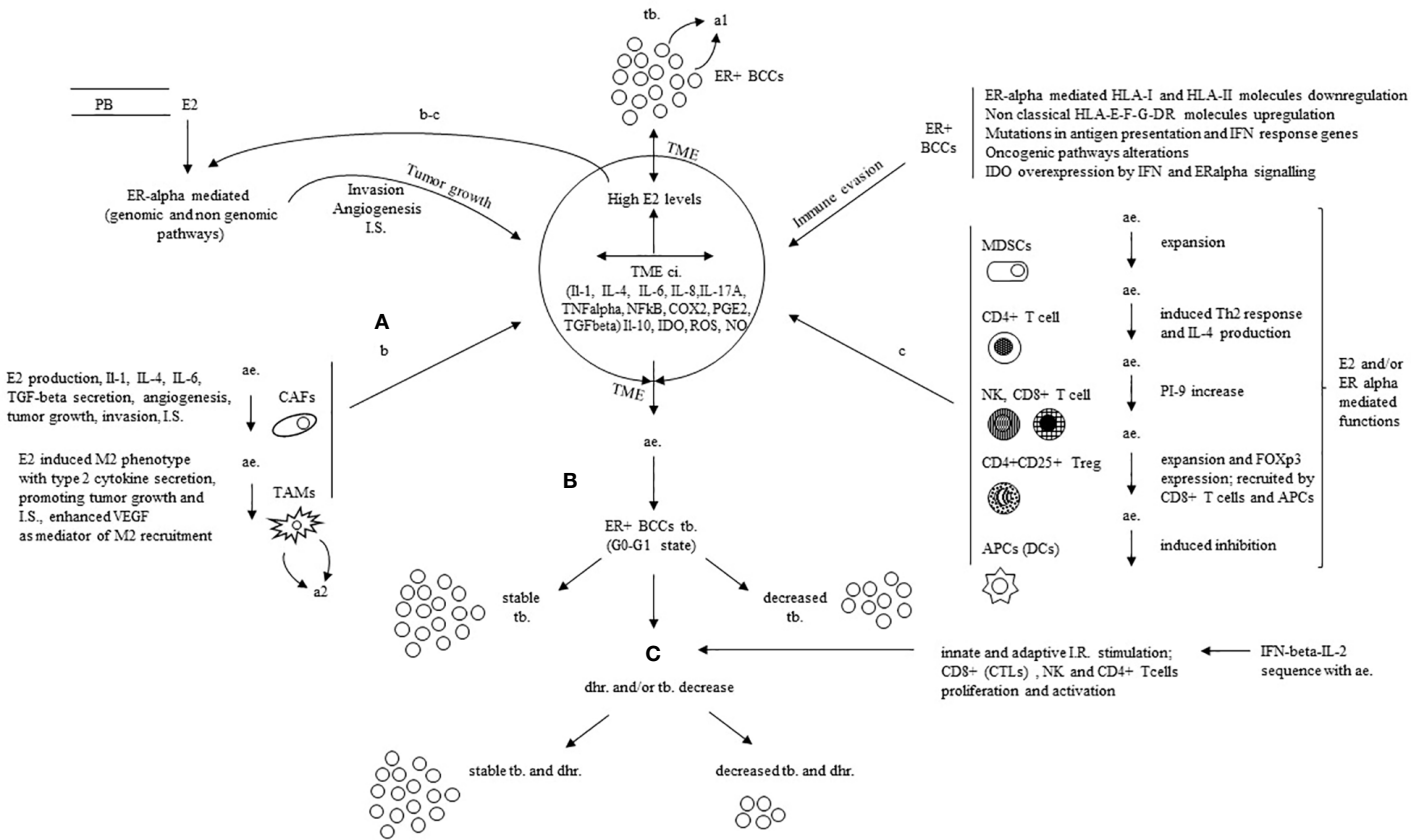
Mechanistic rationale for the successful immune manipulation with the addition of IFN-beta-IL-2 sequence to antiestrogens in ER positive breast cancer. **(A)** E2 from PB (A) and TME (b-c loops) foster tumor growth, invasion, IS. and angiogenesis mostly through ERalpha mediated actions (genomic and non genomic pathways) and some other mechanisms in ER+BCCS; TME, mainly CAFS, TAMs, immune cells and APCs further contribute with production and secretion of inflammatory cytokines (IL-1, IL-4, IL-6, IL-8, IL17A, TGFbeta, TNFalpha, NFKB, COX2, PGE2), suppressive mediators (ROS, IDO, NO, IL-10) as well as other described mechanisms. **(B)** In patients in clinical benefit during ae., the induced G0-G1 state downregulates the ERalpha mediated actions with stable or decreased T.B. **(C)** The addition of IFNbeta-IL-2 sequence to ae. boosts the immune response through induced proliferation and activation of the effector immune cells; it derives a stable t b. with delayed hormone resistance or decreased tb. and delayed hormone resistance. E2, estradiol; ER, estrogen receptor; ac., anti estrogen; ER+ BCCS, ER positive breast cancer cells; PB, peripheral blood; I.S., immune suppression (inclusive of immune evasion and immune inhibition); TME ci., tumor microenvironment chronic inflammation; HLA, human leucocyte antigen; IFN, interferon; IL, interleukin; IDO indoleamine-pyrrole 2, 3 dioxygenase; ROS, reactive oxygen species; NO, nitric oxide; TNF alpha, tumor necrosis factor alpha; TGFbeta, transforming growth factor beta; NFKB, nuclear factor k light-chain enhancer of activated B cells; COX2, cyclooxygenase-2; PGE2, prostaglandin E2; CAFs, cancer associated fibroblasts; TAMs, tumor-associated macrophages; M2, type 2 TAM; VEGF, vascular endothelial growth factor; MDSCs, myeloid-derived suppressor cells; Th2, type 2 T helper cell; PI-9, proteinase inhibitor 9; FOX-p3, forkhead box p3; APC, antigen presenting cell; tb., tumor burden; dhr., delayed hormone resistance; CTL, cytotoxic T lymphocyte; a1, autocrine loop in ER+BCCs sustained by increased TNFalpha-E2 production; a2, autocrine loop in TAMs sustained by E2 increased VEGF; b-c, paracrine loops promoting tumor growth, invasion, angiogenesis and IS (functions additional to those mediated by genomic and non genomic pathways in ER+ BCCs).

In one of our first reports on the proposed hormone immunotherapy in ER+ metastatic breast cancer patients (116), the clinical outcome was correlated with immunological data. It was found that ‘in patients with clinical benefit, eosinophils, total lymphocytes, CD4+, CD8+ and CD16 + 56+ cells significantly increased after interleukin-2 administration (from p<0.012 to p<0.000). In the patients with progressive disease only a slight increase in eosinophils occurred (p=0.038)’ (116). An update of laboratory results during hormone immunotherapy was the subject of two further papers. In the former (115), it was reported that ‘in clinical benefit interleukin-2 administration was followed by a significant increase in total lymphocytes, CD4+, CD8+, CD16 + 56+ (NK) cells, IL-6, IL-12, and C-reactive protein (CRP) (from p<0.04 to p<0.000) but no change in IL-10 and TGF-beta1 was observed. During progressive disease, no change was observed in the former parameters, concomitant with a significant increase in IL-10 (p<0.020) and a significant decrease in TGF-beta1 (p<0.023)’. In the latter (117), with additional data, it was found that ‘during clinical benefit as opposed to progression a significant increase in the total number of lymphocytes, CD4+, CD8+, CD16 + 56+ (NK) cells, CRP and IL-12 was confirmed but not IL-6. At the progression, both basally and after interleukin-2 stimulation the mean values of CD4+CD25+ were more than two-fold higher than during clinical benefit with a decrease of CD4+ plus CD8+ (T effector) CD4+CD25+ (Treg) ratio’. The G0-G1 state has been found as a condition favouring a successful immune manipulation and has been widely discussed by us in recent reviews (134, 138). Furthermore, the primary mechanisms of immune suppression currently acknowledged have been presented in details in **Tables 1A**, **B**, **2**. These immune suppression mechanisms, mediated by oestrogens and ERs in ER+ breast cancer cells as well as other cells in the TME, align with our earlier hypothesised model. This is schematically represented here and is likely down-regulated in patients experiencing clinical benefits during anti-oestrogen treatment (**Figures 1B**, **2B, C**, paracrine loops). This reversion of immune suppression by anti-oestrogens is documented in our study by lower levels of CD4+CD25+ T cells as well as higher IL-12 values during clinical benefit than at the progression. This likely allowed the beta IFN-IL-2 sequence to significantly boost the effector immune cells of the innate and adaptive immune response (**Figures 1C**, **2**) as demonstrated by the highly significant increase in total lymphocytes, CD8+, CD4+ T cells and NK cells reported during the clinical benefit of the studied breast cancer patients (115, 117). More in particular, oestrogens coming from peripheral circulation and secreted in TME by CAFs and TAMs promote tumour growth, invasion, immune suppression and angiogenesis through genomic and non-genomic pathways and the other reported mechanisms in ER+ breast cancer cells; CAFs, TAMs, immune cells and APCs further contribute with production and secretion of inflammatory cytokines (IL-1, IL-4, IL-6, IL-8, IL17A, TGF-beta, TNF-alpha, NFkB, COX2 and PGE2), suppressive mediators (ROS, IDO, NO, IL-10), as well as other described mechanisms (**Figure 2A**). In breast cancer patients in clinical benefit during anti-oestrogens, the induced G0-G1 state likely down-regulates the ER-alpha mediated actions and the tumour burden remains stable or decreases (**Figure 2B**). The administration of IFN-beta-IL-2 sequence in association with anti-oestrogens boosts the immune response by stimulating proliferation and activation of the effector immune cells; as a consequence, hormone resistance is delayed, and again, tumour burden remains stable or decreases (**Figure 2C**).

As expected, the benefit in terms of median PFS and OS time positively correlated with the rate of ER+ breast cancer cells and the type of response to hormone therapy; in fact, it was significantly better in those with higher ER+ rate and more in responding patients than in those in stable disease during hormone-immunotherapy (117, 138). It has been reported that immunity in cancer TME, also termed ‘immunostat’, is tissue-specific; and its regulation as tumour growth is multi-factorial in origin (139). However, in metastatic setting, most target therapies, at best, affect one or a few pathological molecular pathways. This can account for the relatively early development of resistance and the involvement of non-cancerous cells for the concomitant side effects. From this point of view, anti-oestrogens in metastatic endocrine-dependent breast cancer represent a unique model of a single drug addressing multiple pathological immunological and tumour-growth- promoting targets. This likely explains the relatively prolonged clinical benefit without relevant side effects. Our findings suggest that this peculiarity additionally can significantly be improved by a concomitant immune stimulation with IFN-beta-IL2 sequence.

### 5.3 Clinical outcome: aromatase inhibitors combined with cyclin-dependent 4/6 kinase inhibitors and IFN-beta-IL2 sequence combined with conventional anti-estrogens

Recently, the CCND1-CDK4/6-RB molecular pathway has been investigated as a useful target in increasing the clinical benefit of ER+, HER2- breast cancer patients on first-line hormonal therapy; this constitutive pathway controls and governs whether a cell move on or arrests at the G1-S phase (140, 141). In these ER+, HER2- metastatic breast cancer patients, CDK 4/6 inhibitors, namely ribociclib (121), palbociclib (122, 123) and more recently abemaciclib (124), due to significant increase of PFS in randomised clinical trials compared to antioestrogens alone, after FDA approval, have entered into clinical practice. **Table 3** summarises the results reported in these main clinical trials. Median PFS ranged from 25.3 months with ribociclib (121, 125) to 28.2 months with abemaciclib (124). Median OS has not yet been reached in the clinical trial with abemaciclib, while in a more recent evaluation of ribociclib (121, 125) and palbociclib (122, 123) trials, no significant difference was found in the treated patients *vs*. controls for palbociclib (53.9 *vs*. 51.2 months), while it occurred for ribociclib (63.9 *vs*. 51.4 months; P = 0.008). However, in all these trials, grades 3–4 AEs have been reported in > 10% of the patients receiving CDK inhibitors. In our observational 2:1 controls-case study, median PFS and OS were 33 and 81 months, respectively, without any relevant AEs. Overall, these findings suggest that in ER+ metastatic breast cancer patients in clinical benefit, during the first-line salvage hormone therapy, a concomitant stimulation of the effector immune cells is a valid choice to hormone therapy alone. The recently reported reversion of the immune suppressive TME by anti-oestrogens (137) is consistent with our initial hypothesis. In this favourable condition, the increase of the immune response by the INF-beta-IL-2 sequence is a therapeutic intervention involving a physiological mechanism that likely accounts for no important concomitant AEs (18). Differently, the inhibition of the G1-S checkpoint by the CDK 4/6 inhibitors likely involves tumoral and non-tumoral cells with a common occurrence of relevant AEs. Moreover, notably, the cost per patient per year of the proposed immunotherapy with INF-beta-IL-2 sequence is about 8 to 18 times cheaper than that with the CDK 4/6 inhibitors. However, the last but not the least important issue to be taken into careful consideration is that the different rationale suggests a synergic activity of our proposed immunotherapy with antioestrogens and CDK 4/6 inhibitors.

## 6 Conclusions

The reported experimental and clinical data point out that ER+ breast cancer is a molecular subtype where a successful active immune manipulation, favoured by the G0-G1 state induced by anti-oestrogens, can be attained. The proposed strategy with additional INF-beta-IL-2 sequence in endocrine-dependent metastatic ER+ breast cancer patients seem to be effective, at least as effective as CDK 4/6 inhibitors but with a less harmful treatment profile. If these findings are confirmed by a prospective multicentre trial, which is expected in light of the provided data, it is reasonable that the proposed hormone immunotherapy can also be tested in the adjuvant setting of ER+, HER2- breast cancer patients with a high risk of relapse. Additionally, the different rationale suggests a synergic activity of our proposed immunotherapy plus antioestrogen association with the currently recommended CDK 4/6 inhibitors or with PD1/PDL1 inhibitors. Overall, this paves the way for a change in clinical practice in this most common molecular subtype of breast cancer.

## Author contributions

AN: conceptualization, writing, review, and editing; GR: data curation; PF: formal analysis, writing, review, and editing. All authors contributed to the article and approved the submitted version.
